# Skeletal Muscle Cysticercosis of the Calf: A Rare Case

**DOI:** 10.7759/cureus.33290

**Published:** 2023-01-03

**Authors:** Rekha Petkar, Sunil Panchabhai, Kapil V Gothwal, Bhavesh Mahajan, Kinjal Vasava

**Affiliations:** 1 Department of General Surgery, Aundh District Hospital, Pune, IND; 2 Department of General Surgery, Dr. D. Y. Patil Vidyapeeth University, Dr. D. Y. Patil Medical College, Hospital & Research Centre, Pune, IND; 3 Department of General Surgery, Topiwala National Medical College & B. Y. L. Nair Charitable Hospital, Mumbai, IND; 4 Department of General Surgery, Dr. D. Y. Patil Medical College, Hospital & Research Centre, Pune, IND

**Keywords:** taenia solium, cysticercosis, skeletal muscle, albendazole, infectious disease

## Abstract

Cysticercosis in humans is caused by larval cysts of the tapeworm *Taenia solium*. Any tissue in the human body can become infected by these larval cysts, but the central nervous system, skeletal muscles, subcutaneous tissues, and eyes are the most frequently affected. Muscle cysts are uncommon and typically do not present with any symptoms. In this study, we present a rare case of skeletal cysticercosis as a calf swelling and its management.

## Introduction

Human cysticercosis is a parasitic infestation caused by larval cysts of the intestinal cestode *Taenia solium* [1]. The disease known as human cysticercosis is quite common in parts of Africa, Southeast Asia, and Eastern Europe [2]. Infestations are more prevalent in underdeveloped nations due to a mix of rural society, population density, and poor sanitation that increases human-pig contact and increases the likelihood of fecal contamination. Fruit and vegetables that have been fertilized with contaminated human excrement or infected food handlers who neglected to properly wash their hands before handling the food are usually the sources of contamination. Humans are the definitive hosts of *Taenia solium* [3]. When the larvae mature into adult tapeworms, they shed the proglottids into human feces, which can contaminate the pig's food supply. The larvae also penetrate the gut mucosa, enter the bloodstream and lymphatics, and become distributed throughout the body's tissues, including the brain, subcutaneous tissue, lungs, liver, heart, and skeletal muscles. The pigs' ingested eggs mature into the larval stage, pass through the intestinal wall into the bloodstream, settle in various pig tissues, and eventually form *Cysticercus cellulosae*, the encysted larval form. Cysticercosis most usually affects the central nervous system, which is known as neurocysticercosis [4]. Rarely, it may cause the isolated involvement of skeletal muscles, ocular muscles, and subcutaneous tissue. Here, we report a rare case of skeletal cysticercosis presenting as an isolated calf swelling not responding to conservative management.

## Case presentation

**Figure 1:**
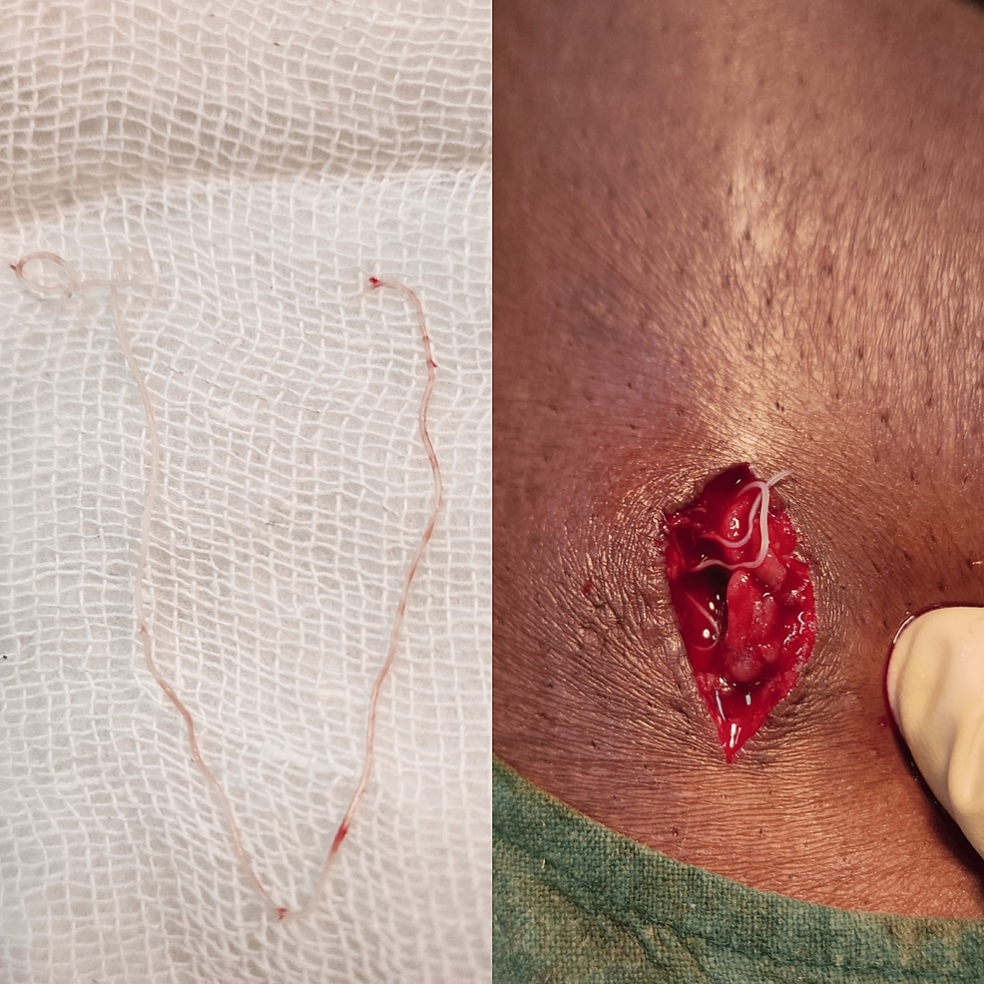
Live worm (Taenia solium).

A 28-year-old male with no known comorbidities came to the general surgery outpatient department with chief complaints of pain and swelling in the left calf region for 30 days. He had no relief even after taking over-the-counter analgesics. In the general surgery outpatient department, he was examined and was found to have swelling of 4 × 3 × 2 cm in the left calf, cystic in consistency, and tenderness was present with no local rise of temperature. He was referred to the radiologist for a soft tissue ultrasound that revealed a 14.6 × 4 mm hypoechoic lesion in the lateral aspect of the left calf with moving linear internal echoes/worm infestation/cysticercosis. He was given the treatment of oral albendazole 400 mg two times a day with nonsteroidal anti-inflammatory drugs for a duration of six weeks, but the swelling did not respond to the treatment. He was advised to get a magnetic resonance imaging of the left calf that revealed the presence of a worm in the calf muscle. The patient was admitted, the biochemical investigations were within normal limits, and he was then taken up for surgery. Intraoperatively, the swelling was excised in toto but accidentally ruptured showing the presence of a live worm (Figure 1).

The patient tolerated the procedure well, and the worm was sent for a histopathological examination that revealed consistent with *Taenia solium*; he was referred to an infectious diseases specialist as he had already taken conservative management for six weeks and was advised suture removal after seven days. During his stay in the hospital, the patient was delighted with the kind of care he received.

Consent

The patient was informed about the case, why it was unique, and the authors' interest in having the case published. He voluntarily gave consent to the authors for the publication of this rare case report with its accompanying image.

## Discussion

In humans, cysticercosis is a common condition. Central nervous system involvement is most frequent. The heart, skeletal muscles, subcutaneous tissues, pleura, and mucous membrane are additional locations that are frequently impacted [5]. Disseminated involvement, where many cysts are present, is the most typical type of involvement. As they have no symptoms, solitary cysts go unnoticed. The location, burden, and host response of the cysts all affect the clinical spectrum of the disease. Isolated skeletal muscle cysticercosis is uncommon. The involvement of the skeletal muscles might range from asymptomatic to mild discomfort and muscle atrophy or hypertrophy [6]. Since cysticercosis of the calf muscles is a very uncommon cause of acute calf discomfort and swelling, diagnosing it can be difficult. In our case, the patient complained of calf pain and swelling, but a local examination revealed nothing other than a localized deep tenderness and solitary cystic swelling in the left calf. Cystic lesions can be visualized using high-resolution ultrasound. Imaging techniques such as computed tomography and magnetic resonance imaging help locate cysts anatomically; computed tomography is sensitive for finding microscopic calcifications, while magnetic resonance imaging is more sensitive since it can distinguish between the cyst and the scolex. The preferred diagnostic technique for visualizing soft tissue cysticercosis is magnetic resonance imaging [7] like in our case that showed the presence of a worm.

Eosinophilia, elevated IgG, and, most importantly, a positive enzyme-linked immunosorbent assay (ELISA) test for IgG antibody against *Taenia solium* are important laboratory findings [8]. Imaging and serological tests assisted in making a correct noninvasive diagnosis of a very rare entity. Similarly, isolated cysts in the biceps, triceps, and masseter muscles have been described in the literature; however, these cases were only discovered after surgical excision such as in our case from the calf muscle with the presence of a live tapeworm. For neurocysticercosis and disseminated cysticercosis, when there is a considerable release of parasitic antigen from dying parasites, the use of steroids in conjunction with the antihelminthic treatment has been advised [9]. However, only oral albendazole therapy is required for isolated lesions. But in our case, although the patient took oral albendazole therapy with nonsteroidal anti-inflammatory drugs, the swelling remained. The cyst was excised, and when the histopathological report confirmed *Taenia solium*, the patient was referred to an infectious disease specialist. Preventive measures in basic sanitation, such as encouraging people to inspect pork, wash their produce thoroughly, cook their meat thoroughly, drink boiled or filtered water, and wash their hands thoroughly before eating, can help prevent and eradicate cysticercosis [10]. These measures were explained to the patient on discharge.

## Conclusions

Cysticercosis in humans is caused by larval cysts of tapeworm. Isolated calf muscle swelling and involvement are uncommon and rarely reported. Proper history, clinical examination, and biochemical and radiological investigations assist in making an accurate diagnosis. It is a preventable disease that can be avoided through enhanced hygiene techniques.
